# Synthesis of 2-(2-Aminopyrimidine)-2,2-difluoroethanols as Potential Bioisosters of Salicylidene Acylhydrazides

**DOI:** 10.3390/molecules15064423

**Published:** 2010-06-21

**Authors:** Markus K. Dahlgren, Christopher T. Öberg, Erika A. Wallin, Pär G. Janson, Mikael Elofsson

**Affiliations:** Department of Chemistry, Umeå University, SE-90187 Umeå, Sweden

**Keywords:** scaffold hopping, Reformatsky reaction, salicylidene acylhydrazide, type III secretion

## Abstract

Salicylidene acylhydrazides are inhibitors of type III secretion in several Gram-negative pathogens. To further develop the salicylidene acylhydrazides, scaffold hopping was applied to replace the core fragment of the compounds. The novel 2-(2-amino-pyrimidine)-2,2-difluoroethanol scaffold was identified as a possible analog to the salicylidene acylhydrazide core structure. The synthesis of a library of 2-(2-amino-pyrimidine)-2,2-difluoro-ethanols is described in this paper.

## 1. Introduction

**Figure 1:**
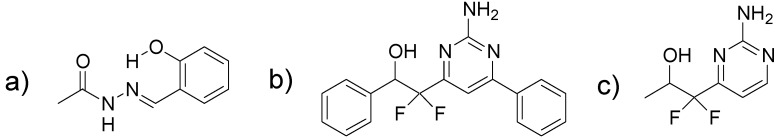
a) The query scaffold of a salicylidene acylhydrazide used for scaffold hopping; b) The highest similarity ranked scaffold, identified in the scaffold hopping program; c) The structure used for a substructure search in SciFinder.

Type III secretion (T3S) is a virulence system found in several clinically important Gram-negative pathogens including *Yersinia* spp., *Chlamydia* spp., *Shigella* spp., *Salmonella* spp., *Pseudomonas aeruginosa*, and enteropathogenic and enterohaemorrhagic *Escherichia coli* [1]. The bacteria use the T3S machinery to secrete and translocate toxins into the cytoplasm of the eukaryotic target cell and thereby create a niche that allows bacterial survival and growth. The T3S system is essential for the bacteria to cause disease and compounds that inhibit T3S have the potential to be developed into novel antibacterial agents [2,3]. Importantly, the T3S machinery can be inhibited without affecting growth and this mode of action is distinct from clinically used antibiotics that all target bacterial growth. Salicylidene acylhydrazides (Figure 1a) were identified in a screen for T3S inhibitors [4] and subsequently it was shown that this compound class blocks T3S in *Y. pseudotuberculosis*, *Shigella flexneri*, enterohaemorrhagic *E. coli, Chlamydia* spp., and *Salmonella* spp. [5,6,7,8,9,10,11,12,13]. While the compounds are interesting as chemical probes to study T3S and clearly indicate a possibility to develop novel antibacterial drugs, the salicylidene acylhydrazide core scaffold suffers from a number of disadvantages. We have found that the salicylidene acylhydrazide T3S inhibitors generally display limited solubility and modest potency. In addition, the scaffold suffers from poor patentability and decomposes rapidly in slightly acidic environments. Salicylidene acylhydrazides have been included in many commercially available screening libraries and as a result the compounds frequently appear in publications and patents. Recently, a strategy based on statistical molecular design was used to compute multivariate quantitative structure-activity relationship models for the salicylidene acylhydrazides [14] and a desirable continuation is to further develop the compounds by modifying the central scaffold. Compounds that mimic and replace the salicylidene acylhydrazide scaffold are of importance since the compound class has delivered biologically active molecules in a number of human and microbial systems as exemplified by several recent publications [15,16,17,18,19,20,21,22,23,24,25,26]. This paper describes the synthesis of a library of 2-(2-aminopyrimidine)-2,2-difluoroethanols, identified from a salicylidene acylhydrazide through scaffold hopping.

## 2. Results and Discussion

### 2.1. Scaffold Hopping from a Salicylidene Acylhydrazide

The global minimum conformation of a salicylidene acylhydrazide (Figure 1a) obtained with Macromodel [27] was used as query scaffold in a scaffold hopping search using SHOP [28]. 

The conformation is planar with an intramolecular hydrogen bond between the phenolic proton and the methylidine nitrogen (Figure 1a). Since the target of the salicylidene acylhydrazides has not been identified, the search was performed at default settings (see Experimental section for details). The reference database used contained 10,556 scaffolds and a total of 124,317 conformers. 2-(2-Amino-6-phenylpyrimidin-4-yl)-2,2-difluoro-1-phenylethanol (**4a**, Figure 1b) was identified as the virtual compound with highest similarity to the query scaffold. A SciFinder substructure search of 2-(2-aminopyrimidin-4-yl)-2,2-difluoro-1-(methyl)ethanol (Figure 1c) (performed January 15, 2010) found no records of compounds with this scaffold. Compound **4a** and a number of analogs were thus targeted for synthesis.

### 2.2. Synthesis of 2-(2-Amino-pyrimidine)-2,2-difluoro-ethanols

**Scheme 1:**
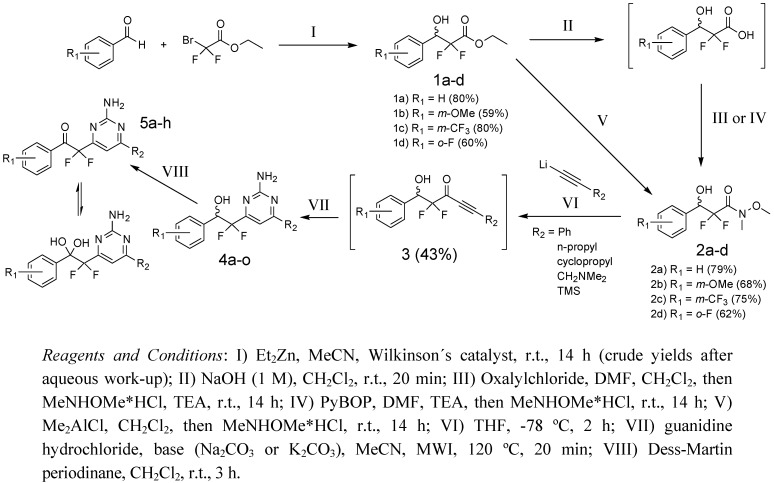
Synthesis of 2-(2-aminopyrimidine)-2,2-difluoroethanols.

Retrosynthetic analysis identified alkynyl ketone **3** (Scheme 1) as a key intermediate, which upon cyclization with guanidine would yield the target compound **4a** following a published procedure [29]. The synthesis of enantiopure alkynyl ketone **3** had previously been described in the literature [30]. This synthetic approach would allow structural variation by selection of a set of benzaldehydes and alkynyl lithium reagents. 

A modified Reformatsky reaction of ethyl bromodifluoroacetate with benzaldehyde gave β-hydroxy ester **1a** in 80% crude yield, as described by Sato *et al*. [31]. The resulting crude β-hydroxy ester was hydrolyzed to the corresponding carboxylic acid and subsequently converted into an acid chloride. Using the acid chloride, the Weinreb amide **2a** was synthesized in 79% yield. Using commercially available lithiumphenylacetylide the Weinreb amide was alkynylated to give **3** in 43% yield following a literature procedure [30]. We unsuccessfully attempted to reproduce the reported yield [30] by first preparing the lithiumphenylacetylide from *n*-BuLi, then from freshly prepared LDA, and finally increasing the amount of lithiumphenylacetylide. Using the protocol published by Tomkinson and co-workers [29] **4a** was obtained in 61% yield (Scheme 1). 

Encouraged by the successful synthesis of **4a**, a small library of analogs was targeted for synthesis. Four different aldehydes were selected based on previous data obtained with salicylidene acylhydrazides [5,14] and used to synthesize the crude products **1a-d**. A fifth aldehyde, nicotinic aldehyde, was also used as starting material but in our hands the Reformatzky reaction failed to produce the target intermediate in acceptable yield. The acid chloride strategy used to prepare**2a **proved hard to reproduce. Using an alternative strategy **2a** and **2c **were synthesized from **1a** and **1c** using the coupling reagent benzotriazol-1-yloxy)tripyrrolidinophosphonium hexafluorophosphate (PyBOP) in 79% and 75% yield, respectively. The coupling reagent *O*-(7-azabenzotriazol-1-yl)-*N,N,N′,N′*-tetramethyluronium hexafluorophosphate (HATU) was found to be as effective as PyBOP (data not shown). These yields were reproducible but to avoid the two-step procedure of ester hydrolysis and amide formation an alternative method using dimethylaluminum chloride to activate the *N,O*-dimethylhydroxylamine, was used to prepare **2a**, **2b**, and **2d** in 78%, 68%, and 62% yield, respectively. The Weinreb amides **2a**-**d** were purified by chromatography and isolated from the corresponding aldehyde in 37–63% yield over two or three steps. Subsequently the Weinreb amides **2a**-**d** were reacted with five different lithium acetylides (Scheme 1, Table 1). 

**Table 1:** The final library of 2-(2-aminopyrimidine)-2,2-difluoroethanols.

ID	R_1_	R_2_	Yield^†^
4a	H	Ph	26%
4b	H	*n*-Propyl	19%
4c	H	Cyclopropyl	11%
4d	H	CH_2_NME_2_	<1%
4e	H	H	8%
4f	*m*-OMe	Ph	25%
4g	*m*-OMe	*n*-Propyl	12%
4h	*m*-OMe	Cyclopropyl	11%
4i	*m*-CF_3_	Ph	42%
4j	*m*-CF_3_	*n*-Propyl	19%
4k	*m*-CF3	Cyclopropyl	24%
4l	*m*-CF_3_	H	28%
4m	*o*-F	Ph	9%
4n	*o*-F	*n*-Propyl	3%
4o	*o*-F	H	12%

^†^Yield starting from Weinreb amides **2a-2d**.

The products obtained using any lithiumacetylide other than lithiumphenylacetylide, rapidly decomposed, even when stored dry in a freezer under a nitrogen atmosphere. Slight decomposition of products was observed even for those products obtained using lithiumphenylacetylide. Therefore the syntheses of compounds **4b**-**n** (Scheme 1, Table 1) were all made from the crude products obtained after alkynylation of **2a**-**d**. For the final cyclization reaction with guanidine four different bases (Li_2_CO_3, _Na_2_CO_3_, K_2_CO_3_, and Cs_2_CO_3_) were tested. There was no significant difference in yield when Na_2_CO_3_ was exchanged with K_2_CO_3_. The cyclization reaction proceeded far slower when Li_2_CO_3_ was used, while reactions performed with Cs_2_CO_3_ resulted in formation of several byproducts. Cyclization with urea and thiourea, using the same conditions as for the guanidine cyclization, was attempted, but the desired oxygen and sulfur analogs could not be obtained. To access additional analogs oxidation of the secondary alcohol, using Dess-Martin periodinane, was performed for some of the compounds (Scheme 1, Table 2). The resulting ketones were in equilibrium with the hydrates (Scheme 1) and purification and characterization proved to be problematic. 

The yields were generally low in the final step, starting from the Weinreb amides (<1%–42%). The poor yields could be attributed to the instability of the alkynyl ketones and that the cyclization reactions had to be performed using crude products. The resulting mixtures after the cyclization reaction contained several impurities and generally required several purification steps, contributing to the poor yields. All target compounds (**4a**-**n**) had a purity of at least 98% as determined by reversed phase HPLC analysis and at least 95% as determined by ^1^H-NMR spectroscopy. 

The final library of products (**4a**-**o**) was tested in a T3S-linked luciferase reporter gene assay in *Y*. *pseudotuberculosis*, essentially as described previously [4]. Only two compounds (**4e** and **4o**) showed effect on the luciferase light emission with 40% inhibition at 1 mM and bacterial growth experiments using *Y*. *pseudotuberculosis* showed that **4e** and **4o** did not inhibit growth at this concentration. 

**Table 2:** The final library of ketones **5a**-**h**.

ID	R_1_	R_2_	Yield^†^
5a	H	Ph	98%
5b	H	*n*-Propyl	73%
5c	H	Cyclopropyl	68%
5d	H	H	27%
5e	*m*-CF_3_	Ph	54%
5f	*m*-CF_3_	*n*-Propyl	55%
5g	*m*-CF_3_	Cyclopropyl	38%
5h	*m*-CF_3_	H	66%

^†^Yield starting from the target compounds **4a-c**, **4e** and **4i-l**.

## 3. Experimental

### 3.1. General

Mass-spectra were recorded with an electrospray Waters Micromass ZG 2000 instrument using an XTerra® MS C_18_, 5 μm particles, 4.6 × 50 mm column and an H_2_O/acetonitrile/0.2% formic acid eluent system. Preparative reversed-phase HPLC was performed on a Beckman System Gold HPLC with a Supelco Discovery BIO Wide Pore C_18_, 5 μm particles, 21.2 × 250 mm column, using an H_2_O/acetonitrile eluent system with or without 0.1% trifluoroacetic acid and a flow rate of 10 mL min^-1^ and detection at 254 nm. Microwave heated reactions were performed in Emrys**^TM^** process vials using a SmithCreator**^TM^** microwave instrument from Biotage. HRMS analysis was performed using a Waters Micromass GCT with an electron impact ion source (EI+), direct inlet (20-400 deg.) or a Bruker microTOF with an ESI ion source in positive mode. ^1^H-NMR and ^13^C-NMR spectra were recorded with Bruker DRX-400 (^1^H-NMR: 400 MHz; ^13^C-NMR: 100 MHz) or DRX-500 (^1^H-NMR: 500 MHz; ^13^C-NMR: 126 MHz) spectrometers. NMR experiments were conducted at 298 K in (CD_3_)_2_CO (residual solvent peak = 2.05 ppm (δ_H_) and 29.84 ppm (δ_C_)), MeOD (residual solvent peak = 3.31 ppm (δ_H_) and 49.00 ppm (δ_C_)), or CDCl_3_ [residual solvent peak = 7.26 ppm (δ_H_) and 77.16 ppm (δ_C_)]. Peak assignments could be established from complementary HMBC, HSQC and COSY experiments. The secondary alcohol proton in the compounds **2a**-**d** and **4a**-**o**, and the amino group protons of compounds **4a**-**o**, and **5a**-**h** could not be identified in many cases due to exchange broadening with the solvent. Equilibrium between the ketones and their respective hydrates (**5a**-**h**) were observed and while the mixtures were pure according to HPLC analysis, interpretable ^13^C-NMR spectra could not be obtained. Therefore only ^1^H-NMR data are given for these compounds (**5a**-**h**). ^1^H-NMR, ^13^C-NMR, are given for all other compounds, except **4d**, that could not be fully characterized due to lack of material. Low resolution electrospray ionization mass spectrometry (ESIMS) data are given for compounds **4a**-**n** and **5a**-**h**. High resolution mass spectrometry (HRMS) data is given for **4o** and the Weinreb amides **2b**-**d**, which did not ionize using the ESIMS instrument.

### 3.2. Scaffold Hopping

Conformational analysis of the salicylidene acylhydrazide was performed using the Macromodel program [27]. Scaffold hopping using the program SHOP [28] at default settings was performed using the global minimum conformation of the salicylidene acylhydrazide as query scaffold. The reference database used was previously built using Maybridge´s building block collection from 2006 and using the virtual reaction manager as described in SHOP [32]. The virtual reaction utility program in SHOP assembles new core structures from fragments of building blocks [32]. The new compounds are expected to be synthetically accessible in five or less steps. The reference database used contained 10,556 scaffolds and a total of 124,317 conformers. 

### 3.3. Synthesis

#### 3.3.1. General procedure for the synthesis of Reformatsky adducts **1a-d**

Wilkinson’s catalyst (0.01 equivalents) was stirred in MeCN (20 mL) under N_2 _atmosphere in an ice bath for 5 min. Ethyl bromodifluoroacetate (1.2 equivalents) and benzaldehyde (8–25 mmol, 1 equivalents) was added and the mixture was stirred for 5 min. Et_2_Zn (1M, hexanes, 1.2 equivalents) was added in portions over 5 min and the reaction mixture was stirred overnight at r.t. The reaction mixture was quenched with excess HCl (1 M) and the organic solvents were evaporated under reduced pressure. The residue was dissolved in EtOAc and washed with HCl (1 M), NaHSO_3_ (aq, sat.), and NaHCO_3_ (aq, sat.). The organic phase was dried with anhydrous MgSO_4_, filtered, and concentrated. The NMR spectrum of the crude product confirmed that the desired β-hydroxy ester had been obtained. The crude β-hydroxy ester was used directly in the next step.

#### 3.3.2. Synthesis of Weinreb amide **2a** via acid chloride formation *in situ*

NaOH (1 M, 40 mL) was added to a separation funnel containing the crude β-hydroxy ester **1a** (1.5 g, 6.5 mmol) in CH_2_Cl_2_ (10 mL), and the funnel was vigorously shaken for 10 min. The water phase was acidified with HCl (1 M) and extracted with EtOAc. The organic phase was dried with anhydrous MgSO_4_, filtered, and concentrated. Most of the resulting crude acid (1.06 g, 5.24 mmol) was transferred to a round-bottom flask and CH_2_Cl_2_ (15 mL) was added. Under stirring in an ice-bath, oxalyl chloride (0.52 mL, 5.96 mmol) and a few drops of DMF was added. After 30 min, pyridine (1.9 mL, 23.5 mmol) and *N,O*-dimethylhydroxylamine hydrochloride (0.62 g, 6.36 mmol) was added, and the reaction was stirred at r.t. overnight under a N_2_ atmosphere. The reaction mixture was diluted with EtOAc, washed with HCl (1 M), and NaHCO_3_ (aq, sat.). The aq. phases were extracted with EtOAc separately. The combined organic phases were dried with anhydrous MgSO_4_, filtered, and concentrated. The resulting oil was purified by column chromatography (heptane:EtOAc 3:1) to give Weinreb amide **2a** in 79% yield (1.02 g).

#### 3.3.3. Typical procedure for the synthesis of Weinreb amides **2a** and **2c**, using PyBOP, exemplified by the synthesis of **2c**

NaOH (2 M, 50 mL) was added to a stirred solution of β-hydroxy ester **1c** (4.38 g, 14.7 mmol) in EtOH (100 mL) at r.t. After 1.5 h, the mixture was acidified using excess HCl (1 M) and the solution was extracted with EtOAc. The organic phase was dried using anhydrous MgSO_4_, filtered, and concentrated. The crude oil was co-evaporated with toluene and stored *in vacuo* before the resulting crude acid was used directly in the next reaction. To the crude acid, *N,O*-dimethylhydroxylamine hydrochloride (1.72 g, 17.6 mmol), PyBOP (9.17 g, 17.6 mmol), and pyridine (3.55 mL, 44 mmol) were added, and the mixture was stirred in DMF (70 mL) at r.t. over night. The reaction mixture was diluted with EtOAc and washed with HCl (1 M) and NaHCO_3_ (aq. sat.). The aq. phases were extracted with EtOAc separately. The pooled organic phases were dried using anhydrous MgSO_4_, filtered, and concentrated. The crude product was purified using column chromatography (heptane:EtOAc 1:1) to give Weinreb amide **2c** in 75% yield over two steps (4.58 g). 

#### 3.3.4. Typical procedure for the synthesis of Weinreb amides **2a**, **2b**, and **2d**, using dimethyl aluminium chloride, exemplified with the synthesis of **2d**

To a solution of *N,O*-dimethylhydroxylamine hydrochloride (1.8 g, 18.5 mmol) in CH_2_Cl_2_ at -78 ºC under N_2_ atmosphere, dimethyl aluminium chloride (22 mL, 0.9 M, heptane) was added and the mixture was stirred for 1 h. Crude β-hydroxy ester **1d** (1.41 g, 5.69 mmol) was added and the mixture was stirred at -78 ºC for 30 min. The reaction mixture was then stirred at r.t. for 5.5 h at which time the β-hydroxy ester had been completely consumed according to TLC analysis. The reaction mixture was quenched with excess HCl (1 M) and diluted with EtOAc. The mixture was filtered through celite, washed with HCl (1 M), NaHCO_3_ (aq, sat.), and extracted with EtOAc. The organic phase was dried with anhydrous MgSO_4_, filtered, and concentrated. The crude product was purified using column chromatography (heptane:EtOAc 4:1 -> 2:1) to give Weinreb amide **2d** in 62% yield (0.927 g).

*2,2-Difluoro-3-hydroxy-N-methoxy-N-methyl-3-phenyl-propionamide* (**2a**). NMR data was in accordance with the data given in the literature [30].

*2,2-Difluoro-3-hydroxy-N-methoxy-3-(3-methoxyphenyl)-N-methylpropionamide* (**2b**). ^1^H-NMR (400 MHz; acetone-d_6_): *δ* 7.30–7.24 (m, 1H), 7.07 (s, 1H), 7.03 (d, *J* = 7.6 Hz, 1H), 6.90 (bd, *J* = 8.5 Hz, 1H), 5.43-5.33 (m, 1H), 5.23 (d, *J* = 5.3 Hz, 1H), 3.81 (s, 3H), 3.80 (s, 3H), 3.24 (s, 3H); ^13^C-NMR (126 MHz; acetone-d_6_): *δ* 163.6, 160.5, 139.3 (d, ^3^*J*(C,F) = 2.7 Hz), 129.8, 121.1, 116.9 (dd, ^1^*J*(C,F) = 258.9 Hz, ^1^*J*(C,F) = 253.9 Hz), 114.7, 114.5, 73.7 (dd, ^2^*J*(C,F) = 26.5 Hz, ^2^*J*(C,F) = 23.0 Hz), 62.3, 55.5, 33.7; ESI-HRMS [M+Na^+^]^+^ calcd. for [C_12_H_15_F_2_NNaO_4_]^+^: *m/z*: 298.0861; found: 298.0861.

*2,2-Difluoro-3-hydroxy-N-methoxy-N-methyl-3-(3-trifluoromethylphenyl)-propionamide* (**2c**). The alcohol proton was exchange broadened. ^1^H-NMR (500 MHz; acetone-d_6_): δ 7.84 (s, 1H), 7.78 (d, *J* = 7.6 Hz, 1H), 7.71 (d, *J* = 7.7 Hz, 1H), 7.63 (t, *J* = 7.7 Hz, 1H), 5.59 (dd, *J* = 17.2, *J* = 7.6 Hz, 1H), 3.82 (s, 3H), 3.26 (s, 3H); ^13^C-NMR (126 MHz; acetone-d_6_): δ 163.1 (bs), 139.1, 132.8, 130.7 (q, ^2^*J*(C,F) = 32.0 Hz), 129.8, 126.0 (q, ^3^*J*(C,F) = 3.7 Hz), 125.4 (q, ^3^*J*(C,F) = 3.6 Hz), 125.3 (q, ^1^*J*(C,F) = 271.2 Hz), 116.6 (dd, ^1^*J*(C,F) = 259.1 Hz, ^1^*J*(C,F) = 254.7 Hz), 73.0 (dd, ^2^*J*(C,F) = 27.1 Hz, ^2^*J*(C,F) = 23.7 Hz), 62.4, 33.4; ESI-HRMS [M+Na^+^]^+^ calcd. for [C_12_H_12_F_5_NNaO_3_]^+^: *m/z*: 336.0630; found: 336.0630.

*2,2-Difluoro-3-(2-fluorophenyl)-3-hydroxy-N-methoxy-N-methylpropionamide* (**2d**). ^1^H-NMR (400 MHz, CDCl_3_): *δ* 7.70–7.55 (m, 1H), 7.40–7.28 (m, 1H), 7.24–7.13 (m, 1H), 7.10–6.99 (m, 1H), 5.71 (bd, *J* = 19.4 Hz, 1H), 3.84 (bs, 1H), 3.75 (s, 3H), 3.26 (s, 3H); ^13^C-NMR (126 MHz; acetone-d_6_): *δ*163.3, 161.3 (d, ^1^*J*(C,F) = 245.8 Hz), 131.1 (d, ^3*^J(C,F) = 8.5 Hz), 130.9 (d, ^3*^J(C,F) = 3.8 Hz), 125.0 (d, ^4*^J(C,F) = 3.8 Hz), 125.0–124.8 (m, 1C) 116.6 (dd, ^1^*J*(C,F) = 259.1 Hz, ^1^*J*(C,F) = 254.4 Hz), 115.6 (d, ^2^*J*(C,F) = 22.6 Hz), 67.1-66.4 (m, 1C), 62.4, 33.6; ESI-HRMS [M+Na^+^]^+^ calcd. for [C_11_H_12_F_3_NNaO_3_]^+^: *m/z*: 286.0661; found: 286.0662. *Tentative assignment.

#### 3.3.5. Typical procedure for the synthesis of pyrimidines **4a-o**, exemplified by the synthesis of **4j**

*n*-BuLi (1.6 M, 3.0 mL, 4.8 mmol) was added dropwise to a stirred solution of diisopropylamine (0.78 mL, 5.6 mmol) in THF at −78 ºC under a stream of N_2_. After 30 min, 1-pentyne (435 mg, 6.39 mmol) was added dropwise. After 1.5 h, a solution of Weinreb amide **2c** (500 mg, 1.60 mmol) in THF (5 mL) was added dropwise at −78 ºC. The reaction was quenched with NH_4_Cl (aq, sat., 5 mL) followed by addition of excess NaHCO_3_ (aq, sat.). The THF was removed under reduced pressure. The remaining aqueous mixture was extracted with EtOAc and the organic phase was subsequently washed with brine, dried with MgSO_4_, filtered, and concentrated. The resulting crude alkynyl ketone was heated using microwave irradiation (120 ºC, 20 + 30 min) with guanidine hydrochloride (457 mg, 4.78 mmol) and Na_2_CO_3_ (677 mg, 6.39 mmol) in MeCN (15 mL). The reaction was filtered and purified by column chromatography (heptane:EtOAc 2:1 -> 1:1) to give pyrimidine **4j** in 19% yield (110 mg) over two steps. 

*4,4-Difluoro-5-hydroxy-1,5-diphenylpent-1-yn-3-one* (**3**). NMR data was in accordance with the data given in the literature [30].

*2-(2-Amino-6-phenylpyrimidin-4-yl)-2,2-difluoro-1-phenylethanol* (**4a**). ^1^H-NMR (400 MHz, MeOD): *δ* 8.17–8.08 (m, 2H), 7.57–7.45 (m, 5H), 7.40–7.28 (m, 4H), 6.49 (s, 2H), 5.49–5.33 (m, 2H); ^13^C NMR (100 MHz, CDCl_3_): *δ* 167.1, 164.7, 164.5 (dd, ^2^*J*(C,F) = 30.7 Hz, ^2^*J*(C,F) = 26.7 Hz), 138.3, 137.8, 131.8, 129.6, 129.1, 129.0, 128.6, 127.9, 119.1 (dd, ^1^*J*(C,F) = 250.4 Hz, ^1^*J*(C,F) = 246.1 Hz), 103.4 (t, ^3^*J*(C,F) = 4.6 Hz), 74.5 (t, ^2^*J*(C,F) = 26.8 Hz); ESIMS *m/z* calcd. [M+H^+^]^+^: 328; found: 328.

*2-(2-Amino-6-propylpyrimidin-4-yl)-2,2-difluoro-1-phenylethanol* (**4b**). The alcohol and amine protons were exchange broadened. ^1^H-NMR (500 MHz; acetone-d_6_): δ7.48–7.44 (m, 2H), 7.39–7.32 (m, 3H), 6.90 (s, 1H), 5.34 (dd, *J* = 17.5, 7.3 Hz, 1H), 2.77–2.68 (m, 2H), 1.74 (app. sextet, *J* = 7.5 Hz, 2H), 0.94 (t, *J* = 7.4 Hz, 3H); ^13^C-NMR (126 MHz; acetone-d_6_): *δ* 170.6, 167.2 (dd, ^2^*J*(C,F) = 31.3 Hz, ^2^*J*(C,F) = 27.9 Hz), 161.1, 137.7, 129.3, 129.0, 128.8, 118.8 (dd, ^1^*J*(C,F) = 251.2 Hz, ^1^*J*(C,F) = 247.3 Hz), 107.1 (t, ^3^*J*(C,F) = 4.2 Hz), 74.4 (dd, ^2^*J*(C,F) = 29.4 Hz, ^2^*J*(C,F) = 24.5 Hz), 37.6, 22.4, 13.7; ESIMS *m/z* calcd. [M+H^+^]^+^: 294; found: 295.

*2-(2-Amino-6-cyclopropylpyrimidin-4-yl)-2,2-difluoro-1-phenylethanol* (**4c**). The alcohol and amine protons were exchange broadened. ^1^H-NMR (500 MHz; acetone-d_6_): *δ* 7.50–7.46 (m, 2H, Ph*H*), 7.39–7.31 (m, 3H, Ph*H*), 6.74 (s, 1H, pyrimidine-C*H*), 5.35 (dd, *J* = 18.1, 6.6 Hz, 1H, benzylic C*H*), 2.23–2.16 (m, 1H, cyclopropyl-C*H*), 1.34–1.27 (m, 2H, cyclopropyl-C*H*_2_), 1.26-1.16 (m, 2H, cyclo-propyl-C*H*2); ^13^C-NMR (126 MHz; acetone-d_6_): *δ* 174.4, 164.8 (dd, ^2^*J*(C,F) = 31.9 Hz, ^2^*J*(C,F) = 26.9 Hz), 160.2, 137.6, 129.3, 129.0, 128.8, 118.6 (dd, ^1^*J*(C,F)= 251.8 Hz, ^1^*J*(C,F)= 247.4 Hz), 104.1 (bs), 74.3 (dd, ^2^*J*(C,F)= 29.7, ^2^*J*(C,F)= 24.3 Hz), 16.4, 13.1, 13.0; ESIMS *m/z* calcd. [M+H^+^]^+^: 292; found: 292.

*2-(2-Amino-6-dimethylaminomethylpyrimidin-4-yl)-2,2-difluoro-1-phenylethanol* (**4d**). The alcohol and amine protons were exchange broadened. ^1^H-NMR (500 MHz; acetone-d_6_): *δ* 7.46 (d, *J* = 7.1 Hz, 2H, Ph*H*), 7.37–7.29 (m, 3H, Ph*H*), 6.96 (s, 1H, pyrimidine-C*H*), 5.33 (dd, *J* = 18.0, 7.2 Hz, 1H, benzylic C*H*), 4.37 (s, 2H, N-C*H_2_*), 2.99 (s, 6H, N-C*H_3_*); ESIMS *m/z* calcd. [M+H^+^]^+^: 309; found: 309.

*2-(2-Aminopyrimidin-4-yl)-2,2-difluoro-1-phenylethanol* (**4e**). The alcohol and amine protons were exchange broadened. ^1^H-NMR (500 MHz; acetone-d_6_): *δ* 8.44 (d, *J* = 5.3 Hz, 1H), 7.46 (d, *J* = 7.1 Hz, 2H), 7.38-7.30 (m, 3H), 6.90 (d, *J* = 5.3 Hz, 1H), 5.36 (dd, *J* = 17.6, 7.3 Hz, 1H);^13^C-NMR (126 MHz; acetone-d_6_): *δ* 165.4–165.0 (m, 1C), 163.0, 158.4, 138.0, 129.1, 129.1, 128.7, 118.8 (dd, ^1^*J*(C,F) = 250.6 Hz, ^1^*J*(C,F) = 246.3 Hz), 107.8 (t, ^3^*J*(C,F) = 4.1 Hz), 74.4 (dd, ^2^*J*(C,F) = 29.5, ^2^*J*(C,F) = 24.4 Hz); ESIMS *m/z* calcd. [M+H^+^]^+^: 252; found: 252.

*2-(2-Amino-6-phenylpyrimidin-4-yl)-2,2-difluoro-1-(3-methoxyphenyl)ethanol* (**4f**). The amine and alcohol protons were exchange broadened. ^1^H-NMR (500 MHz; acetone-d_6_): *δ* 8.12 (bd, *J* = 6.6 Hz, 2H), 7.56-7.48 (m, 3H), 7.30 (s, 1H), 7.28-7.21 (m, 1H), 7.09-7.02 (m, 2H), 6.90-6.84 (m, 1H), 5.43–5.35 (m, 1H), 3.77 (s, 3H); ^13^C-NMR (126 MHz; acetone-d_6_): *δ* 167.2, 164.6, 164.5 (dd, ^2^*J*(C,F) = 30.2 Hz, ^2^*J*(C,F) = 26.6 Hz), 160.4, 139.8 (d, ^3^*J*(C,F) = 1.8 Hz), 137.8, 131.9, 129.6, 129.6, 128.0, 121.4, 119.1 (dd, ^1^*J*(C,F) = 250.1 Hz, ^1^*J*(C,F) = 246.7 Hz), 114.7, 114.5, 103.5 (t, ^3^*J*(C,F) = 4.5 Hz), 74.5 (dd, ^2^*J*(C,F) = 29.3 Hz, ^2^*J*(C,F) = 24.7 Hz), 55.5; ESIMS *m/z* calcd. [M+H^+^]^+^: 358; found: 358

*2-(2-Amino-6-propylpyrimidin-4-yl)-2,2-difluoro-1-(3-methoxyphenyl)ethanol* (**4g**). The amine and alcohol protons were exchange broadened. ^1^H-NMR (400 MHz; acetone-d_6_): *δ* 7.27 (t, *J* = 7.9 Hz, 1H), 7.06–6.99 (m, 2H), 6.95 (s, 1H), 6.91 (ddd, *J* = 8.3 Hz, *J* = 5.6 Hz, *J* = 0.9 Hz, 1H), 5.31 (dd, *J* = 17.3 Hz, *J* = 7.4 Hz, 1H), 3.79 (s, 3H), 2.83-2.73 (m, 2H), 1.81-1.69 (m, 2H), 0.95 (t, *J* = 7.3 Hz, 3H); ^13^C NMR (126 MHz; acetone-d_6_): *δ* 169.9, 167.9 (dd, ^2^*J*(C,F) = 31.6 Hz, ^2^*J*(C,F) = 26.8 Hz), 160.5, 160.4, 139.0 (d, ^3^*J*(C,F) = 1.8 Hz), 129.8, 121.3, 118.7 (dd, ^1^*J*(C,F) = 251.3 Hz, ^1^*J*(C,F) = 247.8 Hz), 114.7, 114.6, 107.1 (dd, ^3^*J*(C,F) = 4.9 Hz, ^3^*J*(C,F) = 3.2 Hz), 74.4 (dd, ^2^*J*(C,F) = 29.4 Hz, ^2^*J*(C,F) = 24.7 Hz), 55.5, 37.0, 22.5, 13.7;ESIMS *m/z* calcd. [M+H^+^]^+^: 324; found: 324

**Figure 2:**
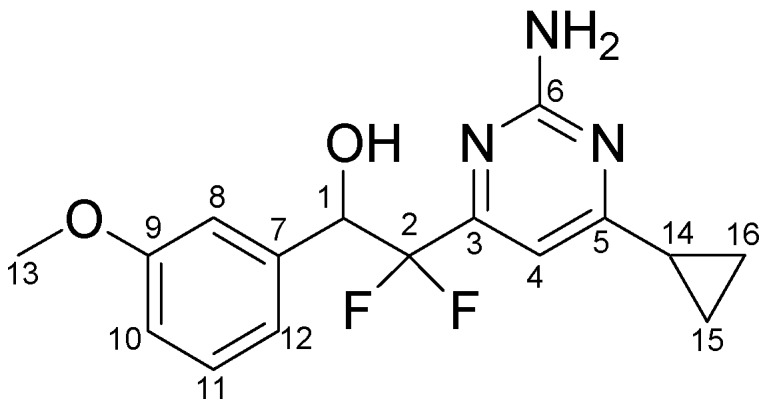
Numbering of carbon atoms of compound **4h**, used for assignment of ^13^C-NMR peaks.

*2-(2-Amino-6-cyclopropyl-pyrimidin-4-yl)-2,2-difluoro-1-(3-methoxy-phenyl)-ethanol* (**4h**). ^1^H-NMR (400 MHz; acetone-d_6_): *δ* 7.24 (t, *J* = 7.5 Hz, 1H, Ph*H*), 7.06–6.99 (m, 2H, Ph*H*), 6.91–6.83 (m, 1H, Ph*H*), 6.72 (s, 1H, pyridimidine-C*H*), 6.21 (s, 2H, N*H_2_*), 5.46–5.40 (m, 1H, O*H*), 5.38–5.28 (m, 1H, benzylic-C*H*), 3.77 (s, 3H, methoxy-C*H_3_*), 1.99–1.89 (m, 1H, cyclopropyl-C*H*), 1.04–0.92 (m, 4H, cyclopropyl-C*H_2_*); ^13^C NMR (126 MHz; acetone-d_6_; for C atom numbering used see Figure 2) *δ* 174.8 (C5), 164.1 (C3, dd, ^2^*J*(C,F) = 31.4 Hz, ^2^*J*(C,F) = 26.8 Hz), 161.5 (C6), 160.4 (C9), 139.3 (C7, d, ^3^*J*(C,F) = 1.8 Hz), 129.7 (C11), 121.3 (C10*), 118.7 (C2, dd, ^1^*J*(C,F) = 251.4 Hz, ^1^*J*(C,F) = 247.1 Hz), 114.7 (C8*), 114.6 (C12*), 104.6 (C4, t, ^3^*J*(C,F) = 4.4 Hz), 74.3 (C1, dd, ^2^*J*(C,F) = 29.4 Hz, ^2^*J*(C,F) = 24.0 Hz), 55.5 (C13), 16.7 (C14), 12.4 (C15*), 12.3 (C16*); ESIMS *m/z* calcd. [M+H^+^]^+^: *m/z*: 322; found: 322. *Tentative assignment.

*2-(2-Amino-6-phenylpyrimidin-4-yl)-2,2-difluoro-1-(3-trifluoromethylphenyl)ethanol* (**4i**). The alcohol and amine protons were exchange broadened. ^1^H-NMR (500 MHz; acetone-d_6_): *δ* 8.17–8.12 (m, 2H), 7.86 (s, 1H), 7.79 (d, *J* = 7.6 Hz, 1H), 7.69 (d, *J* = 7.8 Hz, 1H), 7.62 (t, *J* = 7.7 Hz, 1H), 7.55–7.49 (m, 3H), 7.35 (s, 1H), 5.58 (dd, *J* = 17.8, 6.5 Hz, 1H); ^13^C-NMR (126 MHz; acetone-d_6_): *δ* 167.4, 164.7, 164.0 (dd, ^2^*J*(C,F) = 30.6 Hz, ^2^*J*(C,F) = 26.3 Hz), 139.7 (d, ^3^*J*(C,F) = 1.2 Hz), 137.7, 133.0, 131.9, 130.5 (q, ^2^*J*(C,F) = 31.9 Hz), 129.6, 129.6, 128.0, 125.8 (q, ^3^*J*(C,F) = 3.7 Hz), 125.7 (q, ^3^*J*(C,F) = 3.8 Hz), 125.4 (q, ^1^*J*(C,F) = 271.6 Hz),119.0 (dd, ^1^*J*(C,F) = 250.6 Hz, ^1^*J*(C,F) = 246.1 Hz), 103.4 (t, ^3^*J*(C,F) = 4.4 Hz), 73.8 (dd, ^2^*J*(C,F) = 30.0 Hz, ^2^*J*(C,F) = 24.4 Hz); ESIMS *m/z* calcd. [M+H^+^]^+^: 396; found: 396.

*2-(2-Amino-6-propylpyrimidin-4-yl)-2,2-difluoro-1-(3-trifluoromethylphenyl)ethanol* (**4j**). The alcohol and amine protons were exchange broadened. ^1^H-NMR (500 MHz; acetone-d_6_): *δ* 7.80 (s, 1H), 7.75 (d, *J* = 7.4 Hz, 1H), 7.69 (d, *J* = 7.5 Hz, 1H), 7.61 (t, *J* = 7.6 Hz, 1H), 6.78 (s, 1H), 5.50 (dd, *J* = 17.3, 6.8 Hz, 1H), 2.62 (t, *J* = 7.6 Hz, 2H), 1.74-1.65 (m, 2H), 0.92 (t, *J* = 7.3 Hz, 3H).^13^C-NMR (126 MHz; acetone-d_6_): *δ* 173.3, 164.0 (dd, ^2^*J*(C,F) = 30.6 Hz, ^2^*J*(C,F) = 26.7 Hz), 163.3, 139.5, 133.0, 130.6 (q, ^2^*J*(C,F) = 32.0 Hz), 129.7, 125.9 (q, ^1^*J*(C,F) = 3.7 Hz), 125.6 (q, ^3^*J*(C,F) = 3.8 Hz), 125.4 (q, ^1^*J*(C,F) = 271.3 Hz), 118.8 (dd, ^1^*J*(C,F) = 250.4 Hz, ^1^*J*(C,F) = 246.4 Hz), 106.8 (t, ^3^*J*(C,F) = 4.2 Hz), 73.8 (dd, ^2^*J*(C,F) = 29.7 Hz, ^2^*J*(C,F) = 24.5 Hz), 39.4, 22.4, 13.9.ESIMS *m/z* calcd. [M+H^+^]^+^: 362; found: 362.

*2-(2-Amino-6-cyclopropylpyrimidin-4-yl)-2,2-difluoro-1-(3-trifluoromethylphenyl)ethanol* (**4k**). The alcohol and amine protons were exchange broadened. ^1^H-NMR (500 MHz; acetone-d_6_): *δ* 7.82 (s, 1H), 7.76 (d, *J* = 7.6 Hz, 1H), 7.70 (d, *J* = 7.8 Hz, 1H), 7.62 (t, *J* = 7.7 Hz, 1H), 6.78 (s, 1H), 5.51 (dd, *J* = 18.0, 6.2 Hz, 1H), 2.10-2.06 (m, 1H), 1.16-1.07 (m, 4H); ^13^C-NMR (126 MHz; acetone-d_6_): *δ* 175.4, 163.2-162.6 (m, 2C), 139.5, 133.0, 130.7 (q, ^2^*J*(C,F) = 31.9 Hz), 129.7, 125.9 (q, ^3^*J*(C,F) = 3.7 Hz), 125.7 (q, ^3^*J*(C,F) = 3.6 Hz), 125.4 (q, ^1^*J*(C,F) = 271.3 Hz), 118.6 (dd, ^1^*J*(C,F) = 251.0 Hz, ^1^*J*(C,F) = 246.5 Hz), 105.1, 73.7 (dd, ^2^*J*(C,F) = 30.0 Hz, ^2^*J*(C,F) = 24.4 Hz), 17.1, 11.8; ESIMS *m/z* calcd. [M+H^+^]^+^: 360; found: 360.

*2-(2-Aminopyrimidin-4-yl)-2,2-difluoro-1-(3-trifluoromethylphenyl)ethanol* (**4l**). The alcohol and amine protons were exchange broadened. ^1^H-NMR (400 MHz; acetone-d_6_): *δ* 8.50 (d, *J* = 5.4 Hz, 1H), 7.82 (s, 1H), 7.76 (d, *J* = 7.6 Hz, 1H), 7.71 (d, *J* = 7.8 Hz, 1H), 7.62 (t, *J* = 7.7 Hz, 1H), 6.98 (d, *J* = 5.3 Hz, 1H), 5.52 (dd, *J* = 17.8, 6.5 Hz, 1H); ^13^C-NMR (100 MHz; acetone-d_6_): *δ* 165.5 (dd, ^2^*J*(C,F) = 32.0 Hz, ^2^*J*(C,F) = 26.8 Hz), 162.4, 157.6, 139.3 (d, ^3^*J*(C,F) = 1.5 Hz), 133.0, 130.6 (q, ^2^*J*(C,F) = 32.1 Hz), 129.7, 126.0 (q, ^3^*J*(C,F) = 3.8 Hz), 125.6 (q, ^3^*J*(C,F) = 3.7 Hz), 125.3 (q, ^1^*J*(C,F) = 271.2), 118.6 (dd, ^1^*J*(C,F) = 251.1 Hz, ^1^*J*(C,F) = 246.6 Hz), 107.8 (dd, ^3^*J*(C,F) = 5.3 Hz; ^3^*J*(C,F) = 3.4 Hz), 73.6 (dd, ^2^*J*(C,F) = 30.0 Hz, ^2^*J*(C,F) = 24.3 Hz); ESIMS *m/z* calcd. [M+H^+^]^+^: 320; found: 320.

*2-(2-Amino-6-phenylpyrimidin-4-yl)-2,2-difluoro-1-(2-fluorophenyl)ethanol* (**4m**). The amine and alcohol protons were exchange broadened. ^1^H-NMR (400 MHz; acetone-d_6_): *δ* 8.18–8.11 (m, 2H), 7.77–7.71 (m, 1H), 7.55–7.48 (m, 3H), 7.43–7.35 (m, 2H), 7.29–7.23 (m, 1H), 7.15–7.09 (m, 1H), 5.82 (dd, *J* = 19.5 Hz, *J* = 4.5 Hz, 1H); ^13^C-NMR (126 MHz; acetone-d_6_): *δ* 167.2, 164.9, 164.2 (ddd, ^2^*J*(C,F) = 31.6 Hz, ^2^*J*(C,F) = 25.3 Hz, ^3^*J*(C,F) = 1.9 Hz), 161.5 (d, ^1^*J*(C,F) = 245.4 Hz), 137.9 (d, ^3^*J*(C,F) = 1.8 Hz), 131.8 (d, ^3^*J*(C,F) = 1.8 Hz), 131.0 (d, ^3^*J*(C,F) = 1.8 Hz), 131.0 (d, ^3^*J*(C,F) = 1.8 Hz), 129.6 (d, ^3^*J*(C,F) = 1.8 Hz), 128.0 (d, ^3^*J*(C,F) = 1.8 Hz), 125.5 (d, ^2^*J*(C,F) = 13.5 Hz), 124.9, 119.1 (t, ^1^*J*(C,F) = 248.7 Hz), 115.6 (dd, ^2^*J*(C,F) = 22.6 Hz, ^3^*J*(C,F) = 1.8 Hz), 103.4, 67.5 (dd, ^2^*J*(C,F) = 32.8 Hz, ^2^*J*(C,F) = 23.7 Hz); ESIMS *m/z* calcd. [M+H^+^]^+^: *m/z*: 346; found: 346

*2-(2-Amino-6-propylpyrimidin-4-yl)-2,2-difluoro-1-(2-fluorophenyl)ethanol* (**4n**). The alcohol proton was exchange broadened.^ 1^H-NMR (400 MHz; acetone-d_6_): *δ* 7.70 (bt, *J* = 7.4 Hz, 1H), 7.43–7.36 (m, 1H), 7.24 (td, *J* = 7.5 Hz, *J* = 1.0 Hz, 1H), 7.11 (ddd, *J* = 10.5 Hz, *J* = 8.3 Hz, *J* = 1.1 Hz, 1H), 6.76 (s, 1H), 6.53 (bs, 2H), 5.75 (dd, *J* = 19.1 Hz, *J* = 4.8 Hz, 1H), 2.62–2.57 (m, 2H), 1.70 (h, *J* = 7.5 Hz, 2H), 0.93 (t, *J* = 7.4 Hz, 3H); ^13^C-NMR (126 MHz; acetone-d_6_): *δ* 174.3, 164.5, 163.0 (dd, ^2^*J*(C,F) = 31.6 Hz, ^2^*J*(C,F) = 26.1 Hz), 161.4 (dd, ^1^*J*(C,F) = 245.8 Hz, ^3^*J*(C,F) = 2.7 Hz) 130.9, 131.0, 125.5 (d, ^2^*J*(C,F) = 13.5 Hz), 124.9 (t, ^3^*J*(C,F) = 2.7 Hz), 119.0 (t, ^1^*J*(C,F) = 248.2 Hz), 115.6 (dd, ^2^*J*(C,F) = 22.7 Hz, ^3^*J*(C,F) 2 Hz), 106.6 (t, ^3^*J*(C,F) = 2.7 Hz), 67.4 (t, ^2^*J*(C,F) = 28.3 Hz), 40.4, 22.4, 14.0; ESIMS *m/z* calcd. [M+H^+^]^+^: *m/z*: 312; found: 312

*2-(2-Aminopyrimidin-4-yl)-2,2-difluoro-1-(2-fluorophenyl)ethanol* (**4o**). ^1^H-NMR (400 MHz; acetone-d_6_): *δ* 8.42 (d, *J* = 4.9 Hz, 1H), 7.72 (t, *J* = 7.4 Hz, 1H), 7.44–7.34 (m, 1H), 7.25 (t, *J* = 7.4 Hz, 1H), 7.16–7.06 (m, 1H), 6.85 (d, *J* = 4.9 Hz, 1H), 6.47 (s, 2H), 5.79 (bd, *J* = 19.6 Hz, 1H), 5.50 (s, 1H); ^13^C- NMR (100 MHz; acetone-d_6_): *δ* 164.7-164.5 (m, 1C), 163.1 (dd, ^2^*J*(C,F) = 32.6 Hz, ^2^*J*(C,F) = 25.7 Hz), 161.3 (d, ^1^*J*(C,F) = 245.7 Hz), 160.8, 131.0 (d, ^3^*J*(C,F) = 8.6 Hz), 130.9–130.8 (m, 1C), 125.4 (bd, ^2^*J*(C,F) = 13.7 Hz), 124.9 (d, ^3^*J*(C,F) = 3.5 Hz), 118.9 (dd, ^1^*J*(C,F) = 250.0 Hz, ^1^*J*(C,F) = 245.4 Hz), 115.6 (d, ^2^*J*(C,F) = 22.3 Hz), 107.7-107.6 (m, 1C), 67.6-66.9 (m, 1C); EI-HRMS [M+H]^+^ calcd. for [C_12_H_10_N_3_OF_3_]: *m/z*: 269.0776; found: 269.0773

#### 3.3.6. Typical procedure for the synthesis of ketones **5a-h**, exemplified by the synthesis of **5f**

To a stirred solution of 2-amino pyrimidine **4j** in CH_2_Cl_2_ (1 mL), Dess-Martin periodinane (47 mg, 110 μmol) was added at r.t. After 3 h, the reaction was concentrated and purified by column chromatography (heptane:EtOAc 4:1 -> 2:1) followed by preparative HPLC to give ketone **5f** in 55% yield (11 mg). 

*2-(2-Amino-6-phenylpyrimidin-4-yl)-2,2-difluoro-1-phenylethanone* (**5a**). ^1^H-NMR (400 MHz; acetone-d_6_): *δ* 8.25–8.21 (m, 2H), 8.06–8.03 (m, 2H), 7.71–7.66 (m, 2H), 7.57–7.51 (m, 5H); ESIMS *m/z* calcd. [M+H^+^]^+^: 326; found: 326.

*2-(2-Amino-6-propylpyrimidin-4-yl)-2,2-difluoro-1-phenylethanone* (**5b**). ^1^H-NMR (500 MHz; acetone-d_6_): *δ* 8.00 (d, *J* = 7.5 Hz, 2H), 7.69 (tt, *J* = 7.5, 1.2 Hz, 1H), 7.53 (br t, *J* = 8.0 Hz, 2H), 7.06 (s, 1H), 2.67 (t, *J* = 7.6 Hz, 2H), 1.79-1.71 (m, 2H), 0.96 (td, *J* = 7.4, 0.7 Hz, 3H); ESIMS *m/z* calcd. [M+H^+^]^+^: 292; found: 292.

*2-(2-Amino-6-cyclopropylpyrimidin-4-yl)-2,2-difluoro-1-phenylethanone* (**5c**). ^1^H-NMR (500 MHz; acetone-d_6_): *δ* 8.01 (d, *J* = 7.7 Hz, 2H), 7.68 (t, *J* = 7.4 Hz, 1H), 7.53 (t, *J* = 7.9 Hz, 2H), 7.07 (s, 1H), 2.10–2.08 (m, 2H), 1.10–1.05 (m, *J* = 2.7 Hz, 4H); ESIMS *m/z* calcd. [M+H^+^]^+^: 290; found: 290.

*2-(2-Aminopyrimidin-4-yl)-2,2-difluoro-1-phenylethanone* (**5d**). ^1^H-NMR (500 MHz; acetone-d_6_): *δ* 8.55 (d, *J* = 4.9 Hz, 1H), 8.00 (d, *J* = 7.6 Hz, 2H), 7.71–7.68 (tt, *J* = 7.5, 1.2 Hz, 1H), 7.54 (t, *J* = 7.9 Hz, 2H), 7.12 (d, *J* = 4.9 Hz, 1H); ESIMS *m/z* calcd. [M+H^+^]^+^: 250; found: 250.

*2-(2-Amino-6-phenylpyrimidin-4-yl)-2,2-difluoro-1-(3-trifluoromethylphenyl)ethanone* (**5e**). ^1^H-NMR (400 MHz; acetone-d_6_): *δ* 8.34–8.30 (m, 2H), 8.26–8.21 (m, 2H), 8.08–8.02 (m, 1H), 7.82 (t, *J* = 8.0 Hz, 1H), 7.71 (s, 1H), 7.64–7.61 (m, 1H), 7.58–7.51 (m, 2H); ESIMS *m/z* calcd. [M+H^+^]^+^: 394; found: 394.

*2-(2-Amino-6-propylpyrimidin-4-yl)-2,2-difluoro-1-(3-trifluoromethylphenyl)ethanone* (**5f**). ^1^H-NMR (400 MHz; acetone-d_6_): *δ* 8.30–8.27 (m, 2H), 8.05 (d, *J* = 7.9 Hz, 1H), 7.81 (t, *J* = 7.8 Hz, 1H), 7.05 (s, 1H), 6.33 (s, 2H), 2.65 (t, *J* = 7.6 Hz, 2H), 1.80-1.68 (m, 2H), 0.95 (t, *J* = 7.4 Hz, 3H); ESIMS *m/z* calcd. [M+H^+^]^+^: 360; found: 360.

*2-(2-Amino-6-cyclopropylpyrimidin-4-yl)-2,2-difluoro-1-(3-trifluoromethylphenyl)ethanone* (**5g**). ^1^H-NMR (400 MHz; acetone-d_6_): *δ* 8.31–8.26 (m, 2H), 8.05 (d, *J* = 8.1 Hz, 1H), 7.82 (t, *J* = 8.2 Hz, 1H), 7.09 (s, 1H), 2.11–2.07 (m, 1H), 1.08–1.02 (m, 4H); ESIMS *m/z* calcd. [M+H^+^]^+^: 358; found: 358.

*2-(2-Aminopyrimidin-4-yl)-2,2-difluoro-1-(3-trifluoromethylphenyl)ethanone* (**5h**). ^1^H-NMR (500 MHz; acetone-d_6_): *δ* 8.59 (d, *J* = 5.0 Hz, 1H), 8.32–8.29 (m, 2H), 8.06 (d, *J* = 7.9 Hz, 1H), 7.84–7.81 (m, 1H), 7.16 (d, *J* = 5.0 Hz, 1H); ESIMS *m/z* calcd. [M+H^+^]^+^: 318; found: 318.

## 4. Conclusions

In conclusion, we have demonstrated the synthesis of a small library of 2-(2-amino-pyrimidin-4-yl)-2,2-difluoro-1-(phenyl)ethanols. While the synthetic procedure give low yields in the last step, the yields are generally sufficient to produce enough material for biological screening and evaluation. 

The target compounds **4a**-**o** did not inhibit T3S at concentrations that warrant further investigation. A possible explanation to the lack of activity might be due to that the compounds do not mimick crucial properties of the salicylidene acylhydrazides. The bioactive conformation of the salicylidene acylhydrazides is unknown and different weights could have been applied to the parameters in SHOP if information about the binding had been available. Although the compound databases contained more than 10,556 compounds in around 124,317 conformations, the chemical space sampled might have been insufficient in order to find biologically active salicylidene acylhydrazide analogs. The compounds were evaluated in cell-based assays and even if the compounds mimic the bioactive conformation of the salicylidene acylhydrazides and can bind to the target protein(s), a number of factors, such as low membrane permeability, bacterial efflux, metabolism, or poor distribution of the compounds could still render them inactive. Another factor that might play a role is the metal chelating capacity of the salicylidene acylhydrazides [9], which may be part of the mode of action. The metal chelating ability of **4a-o** is likely to be substantially different. 

The synthesized target compounds **4a**-**o** belong to a structure class that to our knowledge has not been published to date. The compounds have high aqueous solubility compared to the salicylidene acylhydrazides. No precipitation was detected at 200 μM for the entire library and at 1 mM for **4e** and **4o**, whereas many of the active T3S inhibiting salicylidene acylhydrazides start to precipitate at concentrations above 100 μM. The logarithm of solubility in water was calculated, using the program MOE [33], to be around -3 for **4e** and **4o **while as low as -6 for **4i**. The compounds **4a**-**o** all fulfill Lipinski´s rules (molecular weight 251-395, two donor atoms, 4-5 acceptor atoms, and logP 1.6-4.5) and the compounds do not decompose under acidic workup (HCl, 1 M). 

The 2-(2-amino-pyrimidin-4-yl)-2,2-difluoro-1-(methyl)ethanol scaffold is novel and it can be of general interest to include compounds such as the ones presented in high throughput screening collections. Additionally the scaffold can be of special interest in projects where salicylidene acylhydrazides have shown biological activity in mammalian and microbial systems [15,16,17,18,19,20,21,22,23,24,25,26].

## References

[1] Hueck C.J. (1998). Type III protein secretion systems in bacterial pathogens of animals and plants. Microbiol. Mol. Biol. Rev..

[2] Rasko D.A., Sperandio V. (2010). Anti-virulence strategies to combat bacteria-mediated disease. Nat. Rev. Drug Discov..

[3] Baron C. (2010). Antivirulence drugs to target bacterial secretion systems. Curr. Opin. Microbiol..

[4] Kauppi A.M., Nordfelth R., Uvell H., Wolf-Watz H., Elofsson M. (2003). Targeting bacterial virulence: Inhibitors of type III secretion in Yersinia. Chem. Biol..

[5] Nordfelth R., Kauppi A.M., Norberg H.A., Wolf-Watz H., Elofsson M. (2005). Small-molecule inhibitors specifically targeting type III secretion. Infect. Immun..

[6] Muschiol S., Bailey L., Gylfe A., Sundin C., Hultenby K., Bergstrom S., Elofsson M., Wolf-Watz H., Normark S., Henriques-Normark B. (2006). A small-molecule inhibitor of type III secretion inhibits different stages of the infectious cycle of Chlamydia trachomatis. Proc. Natl. Acad. Sci. USA.

[7] Wolf K., Betts H.J., Chellas-Gery B., Hower S., Linton C.N., Fields K.A. (2006). Treatment of Chlamydia trachomatis with a small molecule inhibitor of the Yersinia type III secretion system disrupts progression of the chlamydial developmental cycle. Mol. Microbiol..

[8] Bailey L., Gylfe A., Sundin C., Muschiol S., Elofsson M., Nordstrom P., Henriques-Normark B., Lugert R., Waldenstrom A., Wolf-Watz H., Bergstrom S. (2007). Small molecule inhibitors of type III secretion in Yersinia block the Chlamydia pneumoniae infection cycle. FEBS Lett..

[9] Slepenkin A., Enquist P.A., Hagglund U., de la Maza L.M., Elofsson M., Peterson E.M. (2007). Reversal of the antichlaraydial activity of putative type III secretion inhibitors by iron. Infect. Immun..

[10] Negrea A., Bjur E., Ygberg S.E., Elofsson M., Wolf-Watz H., Rhen M. (2007). Salicylidene acylhydrazides that affect type III protein secretion in Salmonella enterica serovar Typhimurium. Antimicrob. Agents Chemother..

[11] Hudson D.L., Layton A.N., Field T.R., Bowen A.J., Wolf-Watz H., Elofsson M., Stevens M.P., Galyov E.E. (2007). Inhibition of type III secretion in Salmonella enterica serovar typhimurium by small-molecule inhibitors. Antimicrob. Agents. Chemother..

[12] Veenendaal A.K.J., Sundin C., Blocker A.J. (2009). Small-molecule type III secretion system inhibitors block assembly of the shigella type III secreton. J. Bacteriol..

[13] Tree J.J., Wang D., McInally C., Mahajan A., Layton A., Houghton I., Elofsson M., Stevens M.P., Gally D.L., Roe A.J. (2009). Characterization of the effects of salicylidene acylhydrazide compounds on type III secretion in Escherichia coli O157:H7. Infect. Immun..

[14] Dahlgren M.K., Zetterström C.E., Gylfe A., Linusson A., Elofsson M. (2010). Statistical molecular design of a focused salicylidene acylhydrazide library and multivariate QSAR of inhibition of type III secretion in the Gram-negative bacterium Yersinia. Bioorg. Med. Chem..

[15] Manvar A., Malde A., Verma J., Virsodia V., Mishra A., Upadhyay K., Acharya H., Coutinho E., Shah A. (2008). Synthesis, anti-tubercular activity and 3D-QSAR study of coumarin-4-acetic acid benzylidene hydrazides. Eur. J. Med. Chem..

[16] Yang H.Y., Shen Y., Chen J.H., Jiang Q.F., Leng Y., Shen J.H. (2009). Structure-based virtual screening for identification of novel 11 beta-HSD1 inhibitors. Eur. J. Med. Chem..

[17] Liu W.Y., Li H.Y., Zhao B.X., Shin D.S., Lian S., Miao J.Y. (2009). Synthesis of novel ribavirin hydrazone derivatives and anti-proliferative activity against A549 lung cancer cells. Carbohydr. Res..

[18] Vicini P., Incerti M., La Colla P., Loddo R. (2009). Anti-HIV evaluation of benzo[d]isothiazole hydrazones. Eur. J. Med. Chem..

[19] Mircus G., Hagag S., Levdansky E., Sharon H., Shadkchan Y., Shalit I., Osherov N. (2009). Identification of novel cell wall destabilizing antifungal compounds using a conditional Aspergillus nidulans protein kinase C mutant. J. Antimicrob. Chemother..

[20] Romeiro N.C., Aguirre G., Hernandez P., Gonzalez M., Cerecetto H., Aldana I., Perez-Silanes S., Monge A., Barreiro E.J., Lima L.M. (2009). Synthesis, trypanocidal activity and docking studies of novel quinoxaline-N-acylhydrazones, designed as cruzain inhibitors candidates. Bioorg. Med. Chem..

[21] Cosconati S., Marinelli L., Trotta R., Virno A., Mayol L., Novellino E., Olson A.J., Randazzo A. (2009). Tandem application of virtual screening and NMR experiments in the discovery of brand new DNA quadruplex groove binders. J. Am. Chem. Soc..

[22] He L.Y., Zhang L., Liu X.F., Li X.H., Zheng M.Y., Li H.L., Yu K.Q., Chen K.X., Shen X., Jiang H.L., Liu H. (2009). Discovering potent inhibitors against the beta-hydroxyacyl-acyl carrier protein dehydratase (FabZ) of Helicobacter pylori: Structure-based design, synthesis, bioassay, and crystal structure determination. J. Med. Chem..

[23] Patkar C.G., Larsen M., Owston M., Smith J.L., Kuhn R.J. (2009). Identification of inhibitors of Yellow fever virus replication using a replicon-based high-throughput assay. Antimicrob. Agents Chemother..

[24] Marlo J.E., Niswender C.M., Days E.L., Bridges T.M., Xiang Y., Rodriguez A.L., Shirey J.K., Brady A.E., Nalywajko T., Luo Q., Austin C.A., Williams M.B., Kim K., Williams R., Orton D., Brown H.A., Lindsley C.W., Weaver C.D., Conn P.J. (2009). Discovery and characterization of novel allosteric potentiators of M-1 muscarinic receptors reveals multiple modes of activity. Mol. Pharmacol..

[25] Patel V., Mazitschek R., Coleman B., Nguyen C., Urgaonkar S., Cortese J., Barker R.H., Greenberg E., Tang W.P., Bradner J.E., Schreiber S.L., Duraisingh M.T., Wirth D.F., Clardy J. (2009). Identification and characterization of small molecule inhibitors of a class I histone deacetylase from Plasmodium falciparum. J. Med. Chem..

[26] Peterson Q.P., Hsu D.C., Goode D.R., Novotny C.J., Totten R.K., Hergenrother P.J. (2009). Procaspase-3 activation as an anti-cancer strategy: Structure-activity relationship of procaspase-activating ompound 1 (PAC-1) and its cellular co-localization with caspase-3. J. Med. Chem..

[27] MacroModel, version 9.5.

[28] SHOP version 1.0

[29] Bagley M.C., Hughes D.D., Lubinu M.C., Merritt E.A., Taylor P.H., Tomkinson N.C.O. (2004). Microwave-assisted synthesis of pyrimidine libraries. Qsar Comb. Sci..

[30] Iseki K., Asada D., Kuroki Y. (1999). Preparation of optically active alpha, alpha-difluoro-beta-hydroxyketones. J. Fluorine Chem..

[31] Sato K., Tarui A., Kita T., Ishida Y., Tamura H., Omote M., Ando A., Kumadaki I. (2004). Rhodium-catalyzed Reformatsky-type reaction of ethyl bromodifluoroacetate. Tet. Lett..

[32] Bergmann R., Liljefors T., Sorensen M.D., Zamora I. (2009). SHOP: Receptor-Based Scaffold HOPping by GRID-Based Similarity Searches. J. Chem. Info. Model..

[33] Molecular Operating Environment, (MOE), version 2008.10.

